# The long-term psychological processing of an autism spectrum disorder diagnosis in parents

**DOI:** 10.3389/fpsyt.2026.1782789

**Published:** 2026-04-29

**Authors:** Maria Luisa Martino, Laura Vela, Michela Quitadamo

**Affiliations:** 1 Department of Humanities, Federico II University, Naples, Italy; 2 Neuropsychomotor Rehabilitation Center Giffas, Naples, Italy

**Keywords:** autism spectrum disorder, autobiographical memories, diagnosis, narratives, processing

## Abstract

**Introduction:**

A child’s ASD diagnosis represents a critical event for parents, often requiring them to face the loss of their child’s ideal image and reevaluate the family life projects. The aim of this study is to explore how parents retrospectively reconstruct and integrate their child’s ASD diagnosis through autobiographical memories.

**Methods:**

21 parents, 16 mothers and 5 fathers, that received the ASD diagnosis within five years, were administered the Reaction to Diagnosis Interview (RDI). Interviews were audio-recorded, transcribed verbatim and analyzed using a two levels approach. The first one to explore the patterns of meanings that emerged in the whole parents’ autobiographical memories through the Reflexive Thematic Analysis. The second one is to identify patterns of resolution or non-resolution of the impact of the diagnosis.

**Results:**

Findings show suffering and struggling as main themes and subthemes and a prevalence of unresolved diagnoses; gender differences in the way of managing the child-related care tasks, efforts, and coping strategies emerged.

**Discussion:**

In line with literature, our findings suggest that the availability of supportive resources plays a crucial role in facilitating parents’ adjustment and integration of the ASD experience and harmonizing gender differences. They also emphasize that the impact of ASD diagnosis is not a single event but an ongoing process of meaning-making which changes with the child’s developmental path. Our findings highlight the need for cognitive and emotional reconstruction and reframing of parents’ autobiographical memories. These processes play a kay role in shaping how the diagnosis experience is integrated into one’s narrative identity, creating opportunities for transforming the meaning of the remembered experience.

## 1 Introduction

### 1.1 Parents in front of autism spectrum disorder diagnosis: the struggle with processing

According to estimates from the Global Burden of Disease Study 2010, approximately 52 million people worldwide were estimated to be living with a diagnosis of autism spectrum disorder (ASD), with a prevalence of 1 in 132 subjects. Among 8-year-old children in the United States, prevalence estimates have increased from 1.1% in 2008 to 2.3% in 2018, with higher rates observed in males (1, 2). ASD is a neurodevelopmental disorder characterized by the presence of stereotyped, restricted, or repetitive behaviors and the atypical or delayed development of social interaction and communication skills (3, 4). A child’s ASD diagnosis often represents a critical moment for parents and may be experienced as emotionally challenging or distressing. It leads parents to confront the loss of their child’s ideal image and to reevaluate family life projects, expectation and perceived personal limits (5, 6).

In coping with this experience, parents may rely on emotion-focused strategies such as reframing or positive reframing (7–9).

The timing and way the ASD diagnosis is resolved are critical factors in the well-being of parents and their capacity to assume additional caregiving responsibilities. The speed of the diagnostic process, the large number of professionals with whom they must interact in order to obtain a diagnosis, and the knowledge and interpersonal skills of the professionals involved are all critical factors that contribute to the results. These contribute to the processing and integration of the diagnosis (10). A key element in helping parents to accept and process the diagnosis is the time to assess this diagnosis and the type of interaction with the doctor, particularly if it is slow but empathetic and well-communicated (10). The percentage of parents who have resolved the child’s ASD diagnosis tends to be lower in cases of autism. Compared to the rates observed for other conditions, which were 69%, only 33% of mothers reported that their diagnosis had been resolved (11). Recent reviews have highlighted that parental resolution following a child’s diagnosis is a complex and ongoing process involving the gradual adjustment of parents’ expectations and internal representations of their child (12). Resolution is associated with better parental well-being and improved parent–child relationships (13). This difference could be due to factors beyond the child’s symptomatology, such as the heterogeneity of the disorder. While some medical conditions tend to have a more stable or treatable course, ASD is characterized by highly heterogeneous developmental trajectories that are harder to predict (11). These factors can hinder the construction of new parental representations consistent with the diagnosis and psychological adjustment processes. Another important aspect concerns the level of assistance often required by individuals with ASD. Volkmar et al. (14) discovered that 85% of individuals with ASD experience specific care and assistance needs from their parents and families throughout their lifetime. Parents of children with ASD often report higher and more persistent levels of stress over time, as a result of the persistent emotional and behavioral challenges associated with caregiving and the ongoing condition of patients with ASD. This potentially results in long-term consequences for the parents’ mental and physical health.

Additionally, the presence of a child with ASD has a significant impact on not only the physical and emotional levels, but also general goals, family structure and social interactions (15). Mothers are typically the primary caregivers of their disabled children, which results in increased stress for them (16). Studies have shown that the disclosure of an ASD diagnosis can affect mothers’ well-being, including their mental and physical health, their engagement in play, and their behavior with their child (16–19). Fathers, otherwise, report experiencing significantly fewer depressive symptoms than mothers, primarily due to their diminished involvement in the care of their children and, as a result, in their interactions with medical or healthcare professionals. Nevertheless, the extent of their involvement and their collaborative role are still being examined to ascertain whether there has been a change (20). To understand differences in parent-child interactions, Oppenheim (21) highlighted the importance of parental *insightfulness*, defined as the capacity to understand the child’s experiences and actions from their perspective, empathetically accept even challenging or disappointing behaviors, and maintain a multidimensional view of the child. Parents with insightfulness tend to flexibly process and integrate the diagnosis and their own mental representations of themselves and their child and to respond sensitively and appropriately to the child’s signals. Among fathers, *insightfulness* is associated with greater sensitivity in the relationship with their child, while for mothers, it is primarily the resolution of the diagnosis that positively influences parental sensitivity. Greater resolution of ASD diagnosis has been associated with improved attunement and parental insightfulness in the parent-child relationship (13). Additionally, both capacities indicate that parental communication is enhanced, indicating that mentalization is not restricted to the child-parent relationship but also encompasses the marital relationship (21).

Parents of children with ASD are at risk to experience high levels of stress, depression, anxiety, and anger compared to other parents, such difficulties are often linked to the demanding nature of caregiving and the emotional challenges associated with the condition. In this scenario, a protective role is played by the parent’s education level (7, 22–25).

In this context the parent’s ability to manage high levels of stress and adaptation depends largely on the effectiveness and variety of coping strategies implemented and parental self-efficacy (26). Support from family and friends, participation in social support groups, communication with other parents of children with ASD, assistance provided by health and social services professionals, and advocacy activities are the most prevalent coping mechanisms. Faith or religious involvement may also represent an important coping resource for some parents. The utilization of numerous, efficient resources can enhance psychological resilience, reduce the adverse effects of ASD-related stressors, and facilitate a more effective transition to the parenting role (24). Studies have shown differences between mothers’ and fathers’ coping strategies. The first tend to implement emotion-focused coping strategies, such as social and emotional support and spiritual strategies. Nevertheless, fathers exhibited a propensity to employ problem-focused strategies, whereas emotion-focused strategies were implemented primarily to mitigate frustration and prevent family crises. (27).

De Luca Picione et al. (28) describe the development of a “sense of grip” as a meaning-making process through which parents organize their experience of the disorder and adapt to the variability of life context. A person recognizes the necessity of managing the relationship with diseases or disorders by organizing various degrees of flexibility, differentiation, and adaptation to the variability of life experiences and contexts.

### 1.2 The key role of autobiographical memories in critical experiences: a meaning-making process

Understanding how parents process and integrate their child’s diagnosis implies examining how they narrative reconstruct the memories linked to the diagnosis over time. Within a narrative identity framework (29), autobiographical memory (AM), the focus of the present study, plays a central role in the reconstruction of the personal past, connecting past experiences to the present and projecting them into the future as part of an evolving self-story. Autobiographical memory involves recalling life events in narrative form, allowing individuals to organize past experiences from an integrative prospective and construct meaning over time (30–33). Though this narrative structuring of experience, memories and their narration interact in a dynamic and circular process in which meanings are continuously reconstructed (34, 35). This process enables individuals to draw on cognitive and affective information from past experiences and transform them into opportunities for learning and meaning-making (36, 37). Studies show that autobiographical narratives also have a key role in contributing to successful problem solving, coping processes, and pursuing personal goals (38, 39). These kinds of memories serve important directive functions: they help individuals interpret past experiences, guide present decisions, and maintain a sense of self-continuity (40, 41). Autobiographical memories, recalled to the level of awareness, reflect the needs of the self. In fact, on the one hand, they act as moderators of emotional processes; on the other, they play an essential role in protecting and preserving a tolerable self-image to maintain psychological and physical well-being (35, 42, 43). Although previous studies have examined parental resolution of diagnosis in short term, less attention has been paid to how parents narratively reconstruct and integrate this experience in long term through autobiographical memories. In this perspective, autobiographical memories can provide a valuable lens through which to understand how parents reconstruct and make sense of diagnosis of a child with ASD. Therefore, the aim of this study is to explore how parents retrospectively reconstruct and narratively integrate their child’s ASD diagnosis through autobiographical memories. Using the Reaction to Diagnosis Interview (RDI), the study examines patterns of resolution and non-resolution of the diagnosis and the narrative features that characterize parents who have or have not integrated the diagnosis.

## 2 Materials and methods

### 2.1 Institutional review board statement

This study was performed in line with the principles of the Declaration of Helsinki and approved by the Ethics Committee of the Federico II University of Naples, Italy (protocol number 39/2025).

### 2.2 Participants

Twenty-one parents participated in a semi-structured narrative interview based on the Reaction to Diagnosis Interview (RDI) model, including 16 mothers and 5 fathers. Parents’ age was M = 43.33; SD = 7.90; sixteen children were involved, including 3 females and 13 males, with an age of M = 7.8; SD = 2.61. Mothers and fathers were independent cases. The socio-demographic characteristics of the participants are reported in **Table 1**.

**Table 1:** Socio-demographic characteristics of participants.

Participant ID	Parent gender	Age	Educational level	Child gender	Child age	Years since diagnosis	Number of children
1	Female	50	Middle school	Male	10	7	2
2	Male	41	Bachelor’s degree	Male	6	3	2
3	Female	37	High school diploma	Male	6	3	2
4	Female	30	Middle school	Male	7	1	1
5	Male	35	Primary school	Male	7	1	1
6	Female	44	High school diploma	Female	11	9	2
7	Female	44	Bachelor’s degree	Male	9	6	1
8	Female	37	Master’s degree	Male	5	2	3
9	Female	30	Middle school	Male	5	2	1
10	Female	31	High school diploma	Male	7	5	1
11	Female	47	High school diploma	Male	6	3	1
12	Female	50	High school diploma	Male	12	10	1
13	Male	49	Master’s program	Male	10	7	2
14	Male	45	High school diploma	Female	12	9	1
15	Female	47	High school diploma	Female	12	9	1
16	Female	58	High school diploma	Female	9	6	1
17	Male	56	High school diploma	Female	9	6	1
18	Female	45	PhD	Male	6	4	1
19	Female	46	High school diploma	Male	6	3	3
20	Female	39	Bachelor’s degree	Male	4	1	2
21	Female	49	Middle school	Male	10	8	1

The sample is composed by mothers (n=16), while fathers represented a smaller portion of the participants (n=5). The educational level of the parents was generally low to moderate, with the majority holding a high school diploma. The time elapsed since the child’s diagnosis varied across participants with a mean of approximately 5 years (SD = 2.96), indicating that parents were interviewed several years after receiving the diagnosis.

The study took place at a Giffas Neuropsychomotor Rehabilitation Center of the Campania Region, Italy. It is an organization that promotes the social inclusion and empowerment of people with disabilities through a multidisciplinary approach to rehabilitation and rehabilitation processes. The center operates in accordance with the principles of the UN Convention on the Rights of Persons with Disabilities (Law 18/2009), encouraging active participation and full integration into the community. The inclusion criteria were the presence of a child with a formally certified diagnosis of autism spectrum disorder and the child currently being treated at the rehabilitation center where the research was conducted. The child’s current age is no older than 13 years.

Participation was voluntary, following publicity for the study at the referral center, in collaboration with the staff psychologist of the center. Before the interview, each participant was provided with a written informed consent form compliant with the principles of the Declaration of Helsinki (2013) and the Code of Ethics of Italian Psychologists (Article 24).

Data collection took place during September-November 2025 in an *ad hoc* room that guaranteed the confidentiality and privacy of the participants.

### 2.3 Tools

The study used the Reaction to Diagnosis Interview (RDI) (44), a semi-structured narrative interview administered to parents, aimed at exploring their emotional and cognitive reactions to the ASD diagnosis of their child. More specifically, the process of coming to terms with the symbolic loss of the “ideal child” and the prefigured future. This interview allows us to explore how parents integrate the critical event of the diagnosis and the level of adaptation and acceptance achieved with the new reality.

The RDI started from Main’s (1984) (45) studies on the Adult Attachment Interview (AAI), in which the processes of meaning-making of affectively salient experiences can be observed through the way the subject narrates critical events in their relational story. While the AAI explores primary attachment relationships, the RDI allows access to the parent’s mental representations of themselves as caregivers, of their child, and of the diagnosis through autobiographical narratives and the recollection of associated emotional experiences. This highlights any signs of resolution or lack of resolution of the diagnosis experience related to the diagnosis communication. The responses, therefore, may contain elements related to the resolution of the diagnosis or, conversely, lack of resolution. Pianta and Marvin define these two conditions as resolved or unresolved patterns regarding the diagnosis.

Parents who are somewhat detached from the diagnosis experience tend to avoid engaging with what may have been a previous emotional and cognitive state. Others are unable to convincingly describe any progress that may have occurred between their previous and current mental states. Parents who, as we will see, are described as resolved can usually provide detailed descriptions of the diagnosis experience, speaking openly about their own emotional states, both positive and negative, and their evolution. Thus, they can distinguish those past feelings from the current awareness tied to the daily experience of caring for a child with a disability (16).

The average time of the narrative interview was 25–30 minutes, during which the participant was asked five open-ended questions aimed at eliciting parents’ autobiographical memories, emotional reactions, beliefs, and ideas upon learning of their child’s ASD diagnosis. The interviews were conducted by the same psychologist (LV) during an extended traineeship under the supervision of an expert in clinical psychology (MLM); the interviews were conducted in an interactive way, following, in a respectful and supportive conversational climate, the flow of the narrative production of the parents and supporting parents in recalling their autobiographical memories. In some cases, during or at the end of the interview, particularly in the process of recollection of distressing or emotionally painful memories, parents reacted with tears, sadness, and long silences. No parent wanted to interrupt the interview, despite being provided with the opportunity. These reactions were welcomed and contained during the interview, and each parent was offered the possibility to explore in depth some painful aspects of their story with a clinical psychologist of the rehabilitation center.

The interview included the following guiding questions: 1. When did you first realize that your child had medical problems? 2. What did you feel when you realized that your child had medical difficulties? 3. Have those feelings changed over time? If so, how? 4. Tell me exactly what happened when you learned of your child’s ASD diagnosis. Where were you? Who else was with you? What did you think? What did you feel at that moment? 5. Parents sometimes imagine or have ideas about why they have a child with ASD. Do you have any ideas about this, or do you imagine anything? (46).

All participants signed the informed consent form stipulated in accordance with the Code of Ethics of the Italian Psychology Association, the Code of Ethics of the National Order of Psychologists, and the Declaration of Helsinki.

### 2.4 Method of data analysis

This study conducted two levels of analysis. The first level aimed to explore patterns of meanings emerged from the whole parent’s narratives; the second level aimed to explore the parent’s resolution or not resolution of the ASD diagnosis.

#### 2.4.1 First level: reflexive thematic analysis

Reflexive Thematic Analysis (RTA) proposed by Braun and Clarke (47) was chosen by reason of the priority of the researcher’s active role in knowledge production (48) and the accordance with a constructivist and dynamic semiotic theoretical background that guides this study. We maintain that this alignment between the theoretical background of the study and the analysis method selected ensures the way in which the subjectivity and the relationship between subject and context generate meanings in an intersubjective space and culture. RTA respects and expresses this bidirectionality, focusing on the meaningfulness of codes and themes rather than the recurrence (48–50).

This process was performed by two authors (MLM & LV), carrying out a collaborative, reflective, and recursive approach, which sought to highlight those overarching themes that could express the central concerns of the participants’ narrative identities (50). Themes are generated by organizing codes around a ‘central organizing concept’ interpreted from the narratives (48). To empower the confirmability of data analysis, when discrepancies occurred between the two authors, author (MQ), who was not involved in the first phase of the analysis, provided interpretations. Through a peer debriefing and reflexive meeting, the research group reached a consensus.

During the data analysis, credibility was achieved by an involvement, persistent observation, and triangulation of all authors; transferability was achieved by comprehensive and detailed explanations; and dependability was achieved by a rigorous documentation of all aspects related to the research process and phases (51).

The analysis was conducted following a six-step process: (1): Familiarization with the data and construction of notes; (2) Generation of initial codes: the coding process was used to produce interpretative labels attributed to the portions of text that expressed thematic aspects; (3) Theme generation: the coded data were reviewed and analyzed to identify possible combinations according to shared meanings to build themes and subthemes; (4) Review of potential themes: this phase required the researchers to conduct a recursive review of themes reflecting the coded data elements; (5) Definition and naming of the themes: in this phase, the final overarching themes and subthemes were defined that best encompassed the entire text materials; and (6) A triangulated discussion of the results with the other authors to confirm the comprehensiveness and meaningfulness of the thematic analysis.

#### 2.4.2 Second level of analysis: resolved vs. unresolved diagnosis processing

Pianta and Marvin developed a coding system that identified a series of indicators of resolution or non-resolution of the diagnosis. The authors divided the parents into two protocol coding patterns: resolved or unresolved regarding their child’s diagnosis (12, 46).

Parents classified as resolved demonstrate a sufficiently integrated emotional and cognitive processing of their child’s diagnosis. They can recognize the reality of the event and talk about it in a coherent, detailed, and present manner. Their narratives include even painful emotions, such as fear, sadness, or initial confusion. These feelings are placed within a developmental path that allows the parent to clearly distinguish what they felt in the past from how they feel in the present. In this sense, the past is not denied or repressed but rather integrated as part of their family and personal history, redefined in light of their new daily life. Resolved parents also tend to reconstruct the image of their child by consciously incorporating the limitations imposed by the diagnosis, along with their resources and potential (52).

Unresolved parents present narratives characterized by inconsistencies, disorganization, or internal contradictions. Their narratives may appear confusing, fragmented, or difficult to follow, as if the memory of the diagnosis continues to evoke a degree of emotional suffering that hinders a linear narrative. Some parents tend to distort the sequence of events, others struggle to maintain a stable perspective, and still others alternate between minimizing and dramatizing the experience. This may reflect a difficulty regulating the emotions associated with the diagnosis experience, emotions that can remain raw and unintegrated, giving rise to feelings of persistent anger, unresolved grief, despair, or passive resignation. Another portion of unresolved parents appears emotionally detached, as if unable to access their experiences or maintain unrealistic expectations for their child, ignoring or denying the effects of the diagnosis. Finally, some parents show signs of disorientation or confusion, a profile that authors connect, in part, to the presence of previous difficult or stressful experiences that may interfere with the parent’s understanding of the diagnosis. (Please see **Table 1** of **Supplementary Materials** for details of resolved and unresolved subcategories). The interviews were audio-recorded and transcribed verbatim and subsequently analyzed using the Reaction to Diagnosis (RDI) coding system. The second author (LV), a psychologist who had studied the RDI manual and its coding guidelines, conducted the initial coding of the interviews. The coding process was supervised by the first author (MLM), a professor of clinical psychology with expertise in qualitative and narrative methodologies. This process ensured consistency in the application of the coding criteria. The interviews were independently reviewed and discussed by the three authors in order to clarify coding decisions and resolve possible discrepancies until consensus was reached.

#### 2.4.3 Findings

The first level of Analysis through RTA shows four main themes and eight sub-themes as seen in **Table 1**.

#### 2.4.4 The uncertainty

This main theme centers on parents’ emotions and thoughts remembered and lived during the phase before the ASD. This includes the process of comprehension of the diversity of the child and the suspicion that something is wrong in the developmental stages. It begins within the proximal setting of the child’s life: the family and the school. Suspicions and concerns about the child’s differences from other children become increasingly real, moving from a temporary condition to a real and stable one.

##### 2.4.4.1 The maternal sixth sense

The first person to notice that something is wrong with her child is very commonly the mother. Mothers often see signs that others (husbands, grandparents, uncles) tend to ignore or downplay. Mothers feel isolated, their concerns about their child not being validated by other family members. For some mothers, the communication of the ASD diagnosis represents a way to rebuild their self-confidence and regain the trust of other family members.

*…At 4 months … I stimulated him with toys … but he didn’t look at the toys, he looked away and then he didn’t hold my hands … I was perplexed to be honest. Having an older daughter, I could already notice the difference … I just know that something was quite not right. So yes, I noticed it right away…* (ID 1, Mother).

##### 2.4.4.2 The school as first signal context

The school acts as the first true social mirror. In school children’s behavior becomes “standardized” and atypicality emerges through contrast from other children. The first signal is often given by teachers.

*…so actually It all started when he was two and a half because at nursery school … during his second year the teachers felt the child needed to be monitored, observed because he wasn’t up to par with the others. He tended to isolate himself, his gaze was avoidant … then there was the summer, we went back in September and after just a week the teachers told me “we think it would be best to have some evaluations done, because he avoids eye contact and doesn’t speak”.* (ID 8, Mother).

#### 2.4.5 The rupture of diagnosis

The main theme was centered on the phase of communicating an ASD diagnosis, frequently remembered and lived as a sentence, a lightning or a collapse. The diagnosis was a struggle from which there is no possibility of turning back.

##### 2.4.5.1 Loneliness and grief

This subtheme centers on the suffering and grief of parents regarding the communication of diagnosis within the healthcare system. The communication of an ASD diagnosis is remembered as a cold and sterile moment in which the diagnosis becomes an object. Parents remain alone with a paper that contains the truth but offers no emotional safety or recovery.

*…You know … the doctor is just a person, but I should say that we actually changed neuropsychiatrist after that. Not so much because of him as such, but because I associated his face with that day. At a certain point, I felt I needed to start a path with someone who, overall, had not shocked me in that way … The doctor was very blunt, maybe, objectively, that was appropriate, but … I was left there alone … managing all those emotions … I remember I was completely overwhelmed … those first days were all just dark … even now the doctor … I’ve never really liked him much, because he has never gone into depth, he has never done anything to truly understand the problem … he just signs off on the renewals like that, signs the paper and leaves* (ID 18, mother).

##### 2.4.5.2 The search for meaning and causes

This subtheme centers on the memories regarding the search for a meaning and a cause of ASD diagnosis. This effort is aimed at finding and answering the question, Why me?

Parents explore genetics, birth complications, or their fault to make sense of the shock after the diagnosis is received. This is a way to understand why it happened and to give order to the chaos.

*… from around the fifth month of pregnancy I had, let’s say, a somewhat reduced blood flow to the placenta, so I had to undergo some treatment with medication. In fact, D. was born a bit smaller than average in terms of weight. So I don’t know whether, during that stage of development, receiving less … you know, a bit less blood might have had an impact. I don’t know. I really don’t know … We did the tests and genetic exams … everything came back and nothing. It could be, as I said, that he was born a bit smaller and that this affected brain development at that stage, but I don’t know. You see, I really don’t know. Actually, I analyze some things and say, ‘I have this autistic thing too…’ in the end, a lot of things are my fault…* (ID 7, mother).

#### 2.4.6 The changing of parent role

This main theme centers on memories related to the transformations and changes in the parent and the family system that occurred following the diagnosis. The process of coming to terms with the diagnosis seems to inevitably impose a restructuring of one’s role as a parent, both internally and externally. The parents must redefine the image of the child they dreamed of to welcome the real one.

##### 2.4.6.1 The colonization of familiar time

This subtheme centers on memories related to the concrete transformations that occurred following the diagnosis. Daily life and the routines of the family system are disrupted. The needs of the child overwhelm the parents; leisure time disappears, replaced by a therapeutic “roadmap” that often leaves no space for the parent’s individuality and needs.

*…We’ve been coming to this rehabilitation center for 8 years … we started therapy when A was two years old … my life has become a roadmap between speech therapy and psychomotor skills. It was difficult to enter this world: taking the child to therapy, taking him to school, appointment after appointment. At first, I attended most of the visits with my husband, but then I went on my own, because he had to work, so I handled everything myself, the meetings, the discussions with the teachers, with the center, and also with the therapists. In the beginning, we had meetings like the one we’re having now, and most of the time I was there on my own, so…* (ID 21, mother).

##### 2.4.6.2 Surviving for child’s health

This subtheme focuses on parents’ memories and the frustration and stress associated with maintaining their health to support their child’s well-being. Their health is no longer for themselves but to help build their child’s autonomy.

*…I adapted well to my son’s situation, let me adapt well to mine too for a moment … Also because, of course, from what I’ve been told, even if I don’t have symptoms now, some might appear over the years. So I often find myself wondering, asking myself, ‘How long will I be able to keep up with him? What will emerge?…I have to be well for my son’s health* (ID 21, mother).

#### 2.4.7 The relationship losses

This main theme focuses on the parents’ painful memories of the loss not only of the family’s boundaries but also of all the relationships (couple, relatives, friends, etc.) that existed before the diagnosis. Their child’s autism redefines the family’s boundaries, creating new alliances and profound fractures.

##### 2.4.7.1 The conflict in the parent couple

This subtheme focuses on the parents’ couple and the anger between the roles of mother and father. Often, within the couple of parents, there are different speeds and trajectories of processing the ASD diagnosis. Communication with the couple of parents becomes a short circuit where diagnosis becomes a source of conflict rather than unity. Frequently one parent tends to deny, and one parent tends to accept the new condition.

*…You can’t handle having someone with problems around you … if you keep provoking me, you’re not helping me, on the contrary … Because I can find the strength for my son, but not for him as well. I already have to take care of myself how can I also carry him? You’re an adult, you can take care of yourself* (ID 21, mother).

##### 2.4.7.2 Isolation and social stigma

This subtheme focuses on the defenses parents put in place to cope with fear of social judgment (for example, due to crises or stereotypes). This leads the family to isolate itself, creating a “separate world” where only those who experience the same reality can understand and feel the same emotions.

*…We prefer never to go out because if a crisis breaks out it becomes a disaster for us and for him … things are like this, but this isn’t really a way to live, you know what I mean? It may be fine for him, but now I need to take a moment and reorganize myself … I’m so exhausted that even, for example, if a friend says, ‘Shall we go and look around some shops?’ just something recreational I don’t feel like it. But it’s because I’m tired. But I know that’s not right.* (ID 19, mother).

#### 2.4.8 Second level of analysis: pattern of resolution vs. not resolution

**Figure 1:**
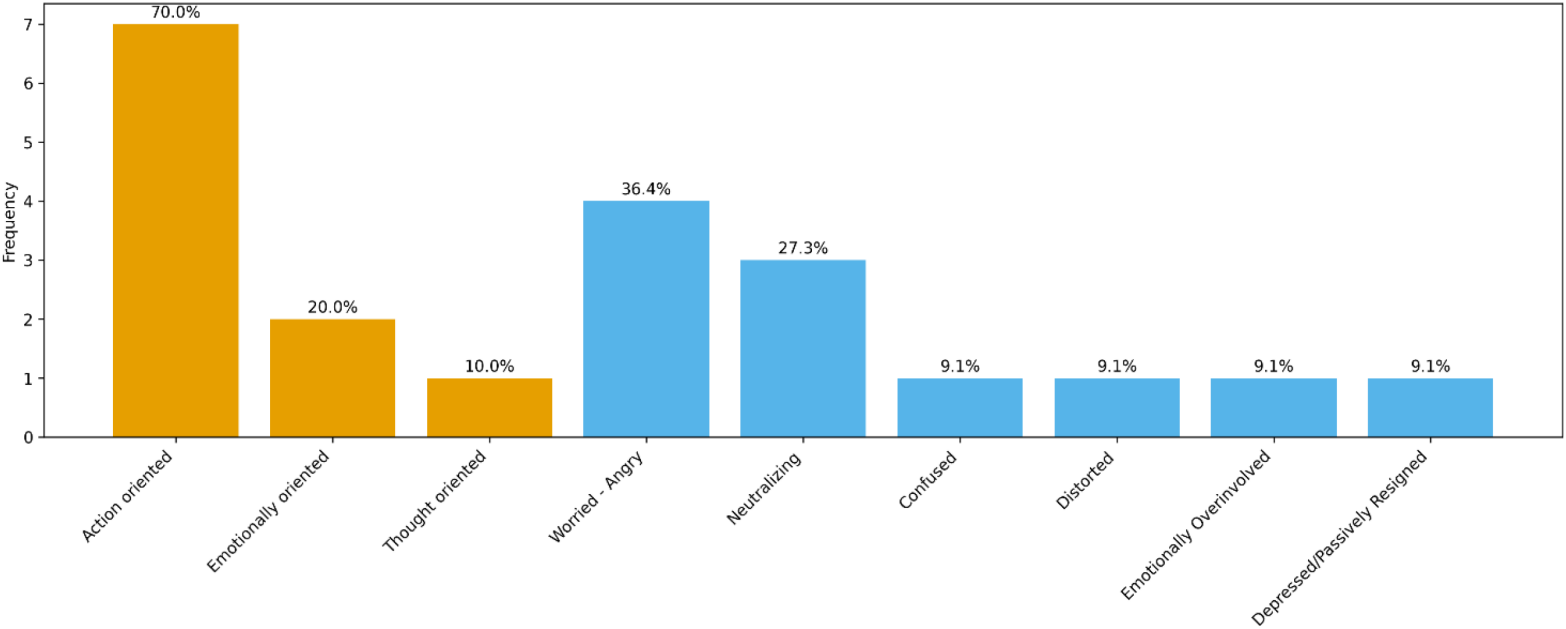
Percentage distribution of subcategories of resolved and unresolved pattern.

Analysis of the RDI indicators allowed us to classify the 21 parents of the sample into two patterns: resolved and unresolved (**Figure 1**). A total of 10 parents (47.62%) were classified as resolved, while 11 parents (52.38%) fell into the unresolved pattern. In line with studies (12), although the distribution is relatively even, a slight prevalence of the unresolved pattern is observed.

As can be seen in **Table 2**, in the pattern classified as resolved (N = 10), the most frequent subcategory was action-oriented (70%), indicating a predominant tendency toward active and pragmatic management of their child’s condition. The emotion-oriented (20%) and thought-oriented (10%) subcategories were less represented, confirming a certain homogeneity in the profile of resolved parents.

**Table 2:** Resolved parents.

Gender	Subcategory	N	% in the group
Women (N = 7)	Action oriented	4	57,1%
	Emotionally oriented	2	28,6%
	Thought oriented	1	14,3%
Men (N = 3)	Action oriented	3	100%

In parents classified as unresolved (N = 11), however, considerable variability in representational subcategories emerged (**Table 3**). The most frequent category was worried/angry (36.4%), followed by the neutralizing pattern (27.3%). The remaining subcategories (confused, distorted, emotionally over-involved, and depressed/passively resigned) were present in only one case each (9.1%), highlighting greater internal heterogeneity compared to the group of resolved parents. This variability confirms the complexity and narrative fragmentation typical of unresolved profiles, with diverse subcategories and no clear predominance of a single representational modality. These results are consistent with the narrative coherence or incoherence of the two patterns.

The distribution of resolved and unresolved patterns was also analyzed separately for mothers and fathers. As previously stated, 16 mothers participated, while 5 fathers participated. **Tables 3**, **4** show the frequency and percentage of the subcategories identified within each group.

**Table 3:** Unresolved parents.

Gender	Subcategory	N	% in the group
Women (N = 9)	Worried – Angry	4	44,4%
	Neutralizing	2	22,2%
	Emotionally Overinvolved	1	11,1%
	Distorted	1	11,1%
	Confused	1	11,1%
Men (N = 2)	Neutralizing	1	50%
	Depressed/Passively Resigned	1	50%

**Table 4:** Reflexive thematic analysis: main themes and subthemes of parent’s narrative autobiographical memories.

Main themes	Sub themes
1 The uncertainty	1.1 The maternal sixth sense
	1.2 The school as the first signal context
2 The rupture of diagnosis	2.1 Loneliness and grief
	2.2 The search for meaning and causes
3 The changing of Parent role	3.1The colonization of familiar time
	3.2 Surviving for child’s health
4.The relationship losses	4.1The conflict in the parent couple
	4.2isolation and social stigma

Seven mothers were classified as resolved, while nine were classified as unresolved. Out of the fathers, three were resolved and two remained unresolved. Action-oriented parents were the most common subcategory for all fathers (100%), and mothers (57.1%). The female group was the only one with the other subcategories. Mothers displayed a diverse range of subcategories among the unresolved parents, whereas fathers were classified into only two groups (neutralizing and depressed/passively resigned).

(Please see the **Table 2** of **Supplementary Materials** to explore the details of resolved and unresolved patterns emerged from the analysis).

## 3 Discussion

Based on the findings of the RTA and the analysis of the RDI patterns, many parents in our sample were classified as unresolved, reporting narratives characterized by disorganization, confusion, and emotional avoidance in long term. This is consistent with the literature, which identifies non-resolution as a common outcome among parents of children with ASD (12). Within the RDI framework such patterns have been widely interpreted as indicative of an ongoing, blocked or fragmented process of meaning-making, in which parents struggle to assign a coherent and cohesive interpretation of the diagnosis. However, several elements that emerged from the qualitative analysis of this group allow us to further explore this process.

A first critical component may pertain to a restricted or fragmented comprehension of ASD. This limitation in knowledge has been associated with a low level of comprehension, which is specifically attributable to cognitive challenges experienced by certain parents. Derguy et al. (53) and Di Renzo et al. (52) have highlighted that the caregiver’s level of education and understanding of concepts related to the diagnosis are factors that facilitate the integration of the problem narratively, thereby enabling the modification and adaptation of one’s life. Of the parents in this sample, nearly one-fifth had only completed middle school education (19.05%), while one parent had only completed primary school.

An additional significant concern that emerged was the significant lack of trust and inconsistency in the rehabilitation therapies that were administered. In fact, certain parents cited treatment approaches that they perceived as ineffective or fragmented, or even shared a narrative of therapists who were not truly aligned with their children. The processing of a diagnosis is impeded by the uncertainty surrounding interventions and the absence of a stable therapeutic alliance, which contributes to elevated levels of stress and a sense of ineffectiveness (54, 55).

Also, the themes of loneliness and the perception of a lack of support, both institutionally and in relationships, are present in both resolved and unresolved patterns in a transversal manner. This absence of support appeared to hinder the process of integrating the diagnosis. In our sample, parents had varying numbers of children, which may influence caregiving demands and the distribution of emotional and practical resources within the family. Recent reviews have highlighted the role of contextual and relational factors in the process of parental adjustment to the diagnosis (12, 13). Although not directly examined in the present study, the presence of multiple children may represent an additional layer of complexity in this process.

The presence of this feeling of loneliness in both categories shows that the daily burden that caregivers continue to bear on account of the absence of an adequate support system does not necessarily decrease as a result of the processing of the diagnosis. These results are in accordance with recent research (22), which demonstrates that the caregiver’s well-being and the stability of the adjustment process are significantly influenced by the quality of the support system, rather than the emotional state of the individual, facilitating resolution, reducing emotional stress, and facilitating a more controlled interpretation of the diagnosis from this perspective.

A final factor that could influence caregivers’ failure to resolve the issue concerns the use of hyper-rationalization as a defensive strategy, with some parents focusing predominantly on technical aspects while marginalizing emotional processing. This cognitive-oriented coping style, rather than integrative, has been associated with difficulties in resolution (17, 44). Overall these findings not only help explain the prevalence of unresolved patterns, but also clarify the marked heterogeneity within this group. The different subcategories of unresolved parents appear to reflect multiple ways of responding to a common underlying difficulty, namely the inability to cognitively and emotionally integrate the diagnosis. Consistent with the literature, this variability does not represent a single dysfunctional pattern, but rather a constellation of responses to a highly challenging experience. These findings highlight the multidimensional and dynamic nature of the adaptation process and the need to conceptualize non-resolution as a complex and heterogeneous outcome.

Regarding resolved patterns, we can observe a greater understanding of their child’s functioning. They show a more accurate representation of their child’s abilities and limitations (16). This ability allows for more adequate emotional regulation and greater attunement to their child’s needs. This competence does not appear to depend solely on educational level but rather on parents’ capacity to observe their child and flexibly adapt to daily needs.

In addition, we observe a greater emotional openness regarding their experiences. Several of these parents discuss their emotions, including negative ones, in a manner that is relatively clear and controlled. The capacity to incorporate one’s own emotional states into the narrative of the experience is one of the most striking indicators of resolution (46).

An additional attribute that is indicative of resolved parents is the cessation of the pursuit of a single or personal cause for their child’s condition. This is a critical indicator of resolution, as it promotes a more comprehensive understanding of the diagnostic reality, interrupts repetitive thoughts, and reduces feelings of guilt. This encourages an increased degree of adaptation and presents the potential for a more adaptable reorganization of the parent’s experience.

The results of this study demonstrate that the quality of available resources, the parent’s capacity to access clear information, the support received, and the coping strategies implemented strongly influence the processes of adaptation to the diagnosis. Non-resolution should not necessarily be interpreted as a sign of individual fragility, but rather as the possible outcome of a complex set of circumstances, such as a lack of institutional support, unmet information needs, loneliness, and severe emotional overload.

The narratives of some parents also suggested elements of positive transformation that have been discussed in the literature in terms of post-traumatic growth (20). In our data, the child’s clinical characteristics did not appear to be the sole determinant of parents’ responses to the diagnosis. Rather, personal meaning-making processes, the availability of social support, and the parent’s capacity to reinterpret the experience seemed to play an important role in how the diagnosis was integrated into their life story. The findings of the present study support the idea that parents’ responses to the diagnosis are not linear or one-dimensional, but it’s a process. Even among parents classified as unresolved, some narratives contained elements of reflection and positive change. This suggests that parents may simultaneously experience aspects of vulnerability and processes of positive transformation, resulting in a dynamic balance that may also be influenced by the quality of the support they receive.

In the group of participants analyzed, therefore, the majority of interviews were conducted with mothers. This discovery, which is not causal, is indicative of a well-documented phenomenon in autism research: fathers are historically underrepresented, poorly involved, or unable to participate in their child’s therapy and care (21). This disparity between mothers and fathers is evident in numerous other studies, including those that examine the relationship between educational attainment or parenting stress and their responses to the diagnosis (22). Consequently, the gender disparities that were observed in our research serve to substantiate, broaden, and contextualize the literature’s depiction: mothers and fathers navigate the diagnosis in distinct manners due to their distinct roles, contexts, and approaches to coping with grief and uncertainty. These discrepancies do not indicate deficiency, but rather two complementary methods of addressing a critical and transformative event such as the diagnosis of autism spectrum disorder.

A further aspect emerging from the findings concerns the role of time in the process of parental integration of diagnosis. In our sample, no linear relationship was observed between the time elapsed since diagnosis and parents’ classification as resolved or unresolved. While some parents reported a coherent integration of the diagnosis even a few years after its communication, others continued to expresso confusion, denial, or emotional disorientation despite a longer temporal distance. This suggests that time alone does not represent a sufficient or reliable factor in facilitating resolution.

These findings are partially consistent with previous research which highlights the dynamic nature of the adaptation process. For instance, some longitudinal studies have shown that parents may move from non-resolution to resolution over time (54), while others remain unresolved even after several years. ASD diagnosis appears not simply a function of time, but rather a complex process influenced by meaning-making, contextual resources, and the quality of the diagnosis and supportive experience (10).

## 4 Clinical implication, limitation and conclusion

Although our investigation is limited by the absence of external quantitative measures, the context-dependent findings, and the small sample size, the importance of providing parents with psychological-clinical, social, and institutional support is underscored. They also need spaces for cognitive and emotional processing, clear informational support, relational context, educational programs, and service continuity. Our findings are consistent with the literature, highlighting that a long-term supportive environment is crucial for the progression of adjustment and integration into the ASD experience and to reconcile gender disparities Furthermore, our research underscored the significance of cognitive and emotional reconstruction and reframing of parents’ memories. As shown, the narrative of AM starting from the ASD diagnosis plays a key role. It could potentially influence the way in which the experience is stored in one’s identity, thereby facilitating the transformation of the meaning of the remembered experience.

Understanding and integrating an ASD diagnosis is a process that requires time, support, and a clinical environment that is psychologically supportive, rather than a single event. This allows the parents to put into words suffering and struggling emotions following the child’s developmental path. The psychological processing of ASD diagnosis is not guaranteed by the time that has passed since the diagnosis. It generates new internal demands and requirements that are linked to the child’s development, ongoing therapies, and expectations for the future.

## Data Availability

The raw data supporting the conclusions of this article will be made available by the authors, without undue reservation.
