# Supplementary material for: The long-term psychological processing of an autism spectrum disorder diagnosis in parents
Source: Front Psychiatry. 2026 Apr 29;17:1782789. doi: 10.3389/fpsyt.2026.1782789 (PMC13168189; doi:10.3389/fpsyt.2026.1782789)
Supplement: Supplementary file 1 [file Table1.docx]

1. Resolved – Action-Oriented

As previously highlighted by theory, this category includes parents who appear to process the diagnosis primarily through action. Their narratives frequently include references to concrete and operational aspects of caring for their child, including what needs to be done, the decisions to be made, and the practical organization of daily life. The process of adapting to the diagnosis, in these cases, therefore revolves around the ability to structure caregiving routines appropriate to the child's needs and to quickly seek resources, interventions, or specialized support.

Among our group of participants, parents classified as action-oriented represented the largest number, with seven parents (four women and three men) out of a total of ten classified as resolved. Their interviews present a generally linear narrative, with an emphasis on the steps taken after the diagnosis was communicated. There is an awareness of initial emotions, but not a blockage of them. These parents tend to focus their narrative on what can be done for their child, thus transforming the diagnosis into an operational starting point. These parents often portray themselves as active and responsible, which allows them to channel concerns or obstacles into concrete actions. This orientation does not imply a reduction in emotional complexity, but rather highlights a mode of regulation in which action becomes the primary means of processing.

The first narrative excerpt that will be presented is taken from the interview of a father classified as resolved and action-oriented. This narrative highlights some characteristic traits of this subcategory: the father's ability to grasp and value his child's progress, a projection toward a realistic yet hopeful future, and a search for new ways of sharing in the parent-child relationship. The narrative thus shows how processing the diagnosis translates into an active orientation, focused on developmental possibilities and the construction of shared meanings.

*[..] Or he gets to certain things and I say, "He did something." Then this fills me with pride, I understand, the pride I was looking for in other things, I get it there. If I see him draw a picture and do it well, color it well, and do it, I say, "That's beautiful." Maybe I expected it when he was 4, now he's 6, but what's the problem? It's not a question of time or age, the important thing is that he got there, so I'm proud of this. I understand that I'm strong, I take all the best I can while I can. Then maybe who knows, but who's to say that one day my son won't be passionate about something that could still open up a path for me to something new [...] my great fear was precisely that, not having the opportunity to share, to understand it, you know [...] Then the great hope that exists is that I see margins that weren't there before. Maybe before, I saw a wall in front of me, but now I see that little by little this wall is starting to peel away some bricks, so it means that the path forward is possible... that the light is there, the rays are shining, so there is hope. This is what gives me so much strength. (Father, ID 2.)*

This excerpt therefore highlights how action orientation is not simply an operational mode but rather a specific form of emotional processing: pride in their child's progress, the search for future possibilities, and the desire to build new forms of sharing represent signs of integration of the diagnosis. The father includes and acknowledges his child's difficulties at different times but places them within a framework of meaning that allows him to transform the initial feeling of confusion into a path of growth.

2. Resolved – Emotionally oriented

This subcategory includes parents who process the diagnosis primarily through the recognition, expression, and sharing of their emotional experiences. Indeed, in their narratives, it is possible to clearly identify feelings such as fear, sadness, joy, hope, or confusion, but these parents describe and integrate them without impeding their ability to reflect on the experience. In this subcategory, as in the previous one, the parent's narrative appears overall clear, coherent, and well-organized.

In the group of participants in this study, only two mothers fell into this subcategory, suggesting that explicit emotional processing can be a less frequent modality than other forms of resolution. Precisely because of their specificity, these narratives therefore offer a valuable perspective on the ability to integrate personal experiences into one's child's diagnostic history.

The narrative excerpt that will be presented will highlight how emotion is recognized, accepted, and gradually transformed by the parent, serving as the starting point for building a new balance in the relationship with the child.

*No, at the beginning it was a blow, a real blow, very heavy […] the fear of the future. And mine… the first thing I thought about was school, what will it be like when he grows up? Because when he's little, you're there for him, so you can help. But when things get more… then you hear a lot of things, bullying, then when you think about it, you say, "Mom, what's going to happen?" I feel bad too, but I say for me the important thing is that he, even if he does it his own way, does it. This. But it's hard every time, every time you have to face a reality, it's hard. But slowly, slowly, as time passes, as years go by, you become more and more involved, so then everything becomes normal [...] I remember one year he did that play, and my husband came in, and he saw him on stage jumping, doing things that an autistic child does anyway. He ran away... and I said, "What if I run away too? What do we do?" And I stayed there, like that, and I moved on.*

*(Mother, ID 9)*

In this narrative, it's possible to see how the resolution emerges through the mother's ability to openly acknowledge her fear and place it within a temporal process, starting from a traumatic beginning, which she herself described as "a blow," followed by a slow adaptation that allowed her, over time, to make the experience "more normal." The moment of acting out constitutes a very powerful symbolic image that further enhances our understanding of this subcategory. The mother consciously chooses to stay, not to withdraw emotionally, integrating the pain and maintaining her presence in the relationship with her child, shedding light on how processing occurs through the acceptance of emotional experiences, which have the potential to become a resource for reattributing meaning to the experience in diagnosis.

3. Resolved – Thought-Oriented

This subcategory includes parents who process the diagnosis through a purely reflective and cognitive approach. Their narratives clearly emphasize thought processes, the attribution of meaning, the active understanding of the condition, and the building of connections between it and their feelings. These parents are characterized by an active search for information about their child, their disability, and what may contribute to their well-being.

In our group of participants, only one mother fell into this subcategory. Her narrative suggests a processing approach focused precisely on understanding her son's functioning, analyzing clinical aspects, the present and future implications of the diagnosis, and a practical organization of daily life. The emotional register is present but somehow filtered by a mental process that allows us to attribute meaning to the experience, maintaining an orderly and reflective approach. Her narrative demonstrates a process focused on identifying a path forward for her son, managing concerns about the future, and distinguishing between the concept of problem and condition, which allows her to give a more stable meaning to the experience.

*Well, the initial moment was obviously one of total confusion, but mostly because we couldn't easily find anyone who offered therapy, even privately. So the main problem was precisely establishing a path forward, which fortunately has stabilized here, so much so that this is the third year we've been attending the center, so we have a certain continuity. […] Yes, at the beginning, it was shocking because you don't know what to do. Everything you do is wrong, it's frustrating, and then obviously, the moment you get the diagnosis, the worry about what will happen after us kicks in, and this thing hit me like a shock wave, and obviously the problem isn't solved because we don't know what will happen after us, but for now, let's say we also have to focus on the days we live in, and therefore... we trust that there will be collaboration from the institutions in the future, even if I want to say here... it's not like you can pray for miracles, I've never believed in miracles, so... let's say that by attending certain contexts, we hope to find the best alternative to the presence of parents. Simple This is definitely it. [...] what was the main problem and seemed insurmountable, and maybe in some ways it is and we pretend it isn't, has become a condition. Because I normally associate the problem with a transitional period in my life, the condition... it's like when a parent dies, there's a new family structure, the problem is when they're ill, the new condition is when they're not there and someone has to fill that role. Right now, our condition is that of caregivers as well as parents, and this is basically what I personally am learning to accept. Because then every day you learn a little, then sometimes... a bit like them, sometimes they take steps forward, sometimes they take steps back. We parents are the same and our progress depends a bit on their state.*

*(Mother, ID 18)*

As can be seen in this narrative, resolution is expressed through intense thought. Indeed, the first step described by the mother concerns her own search for a structured solution to the confusion she experienced. The emotional component is therefore present and acknowledged, but it is immediately linked to reflections on meaning, thus demonstrating a processing method in which thought serves as a tool for emotional regulation and orientation. This allows the mother to understand, define, organize, and name what is happening, thus enabling her to maintain a sense of continuity and competence, integrating the diagnosis within a coherent narrative framework.

1. Unresolved – Worried – Angry

The first subcategory to be addressed among parents classified as unresolved is worried – angry, characterized by a narrative mode in which the diagnosis is approached with intense emotional activation, dominated primarily by feelings of anger, frustration, and intense worry. Parents who fall within this subcategory tend to express unintegrated emotions, often attempting to involve the interviewer in their protest or suffering, thus triangulating the pain. The narratives therefore appear charged with emotional tension, and it is possible to observe the parent's difficulty organizing the experience into a coherent framework.

Among the participants in this study, four mothers, compared to the 11 parents classified as unresolved, fall within this subcategory. Their narratives are characterized by feelings of emotional oppression, feelings of injustice, intense concern for their child's future, and a sense of loneliness that fuels anger. These parents' difficulty mentally processing the diagnosis is expressed through their narrative, which oscillates between a strong sense of mistrust, the need to denounce their injustices, and profound emotional confusion. The excerpt presented below illustrates how these parents tend to let worry and anger dominate their conversation, thus leading to intense and poorly integrated emotional involvement.

*[…] the only fear, as I said, is always what happens next. But now, rightly, I'm all over my daughter because my daughter isn't pro... she's semi-verbal now. Because now, slowly... because when she experiences something, or some pain, I'm always afraid because she now calls me "hurt," but in any case, I always have to figure out where the pain is. So, always the worry. In fact, when she had the epileptic seizure, I did a series of tests, and from there you can really understand the pain, but now... because now I'm more ready. Honey, you have to get your balls out. If you don't get them out, you're no good. I waged war with the world, and I'm still waging war for my daughter. But in the meantime, I have my results. She's doing it. I... I'm going... I'm sick of killing someone for... to do 30 years in prison, but I'm going to prison with pride. For my daughter, justice comes first. Always.*

*(mother, ID 6)*

In light of what was stated previously, this excerpt allows us to focus on the distinctive features of the worried-angry mode. This parent's narrative is, in fact, dominated by emotions intense and poorly elaborated, where worry intertwines with anger, thus giving rise to a combative and polarized tone, with a very strong identification with one's daughter. The reference to prison itself highlights a difficulty in mentalizing the anger, which is acted out at a narrative level rather than actually thought. At the same time, the invocation of justice indicates an attempt to attribute an ethical and moral meaning to one's struggle, thus involving the interviewer as an ally.

2. Unresolved – Neutralizing

The neutralizing subcategory, according to the authors, is characterized by a narrative mode in which the experience of diagnosis is recounted in detail and often comprehensively, but without explicit recognition of negative emotions. The parent describes the events and stages of the diagnostic process in a linear and rational manner, while constantly maintaining an emotional distance that prevents access to the underlying emotional experience. According to the literature, such parents may indeed demonstrate a lack of memory of the emotion experienced in connection with the diagnosis.

Within our group of participants, three parents (two mothers and one father) fell into this sub-category. Their narratives, in fact, show a strong tendency to focus on facts: clinical appointments, assessment results, differences between possible diagnoses, their child's behavioral or language development. Reading these interviews reveals the parent's tendency to construct a logical sequence of events and their child's possible developments, while the emotional impact of the experience remains in the background and unexpressed.

The excerpt presented illustrates precisely this descriptive-rational approach, characterized by a gradual familiarization with the concept of diagnosis, but devoid of explicit references to negative emotions.

*It was, in any case, an ongoing process; there wasn't a "he's autistic," there was a process of... familiarizing oneself with the concept. So, since he... "Oh God, maybe it's a language delay," then a year goes by, two years go by, three years go by, and it can no longer be a language delay. So now that we took the test at the hospital, "the child is on the spectrum," it didn't seem like such big news to me. We already knew, we just wanted to understand... what we wanted to understand was whether he was thought to have an intellectual disability, so this was... for me, he didn't have... but you know, it's hard for a mother to think, "Oh God..."; the father was more worried about this. When they told us, "No, the child is absolutely cognitively normal," I still breathed a sigh of relief. I said, "Well, we can work on it, we have to work on it, let's hope for the best." But, you know, it wasn't a shock because we got there little by little. First visit, second visit, gradually you become familiar with the concept and then, so to speak, you understand it, you start to learn more, you watch videos of other parents, so basically we gradually embraced what was happening without…*

*(mother, ID 8)*

This narrative allows us to observe the neutralizing process in an emblematic way, as the experience is acknowledged by the mother through a sequence of diagnostic steps in which she describes what happened without exploring what these events meant for her. The absence of emotional references, the minimization of impact, and the insistence on technical information, present throughout the interview, reveal a processing process that remains at a cognitive-descriptive level, preventing contact with the most painful emotions.

3. Unresolved – Depressed – Passively Resigned

The subcategory "depressed - passively resigned" is characterized by a narrative style marked by tones of sadness, resignation, and reduced emotional vitality. The parent appears somehow entangled in the diagnostic experience, which is, however, experienced passively, without any elements of hope or developmental possibilities emerging. The interview therefore often takes on a flat tone, but before becoming more intense, it is marked by a strong involvement in the child's care, sometimes experienced as an all-encompassing task.

Only one father fell into this sub-category. His narrative presents a calm but strongly passive tone; the experience of the diagnosis is not described as a source of emotion, but rather as a set of tasks to be faced. The focus is therefore exclusively on what must be done, while the emotional experience remains inaccessible or denied. It might seem that the diagnosis has been accepted, almost as if it were classified as action-oriented due to this particularly pragmatic focus, but in reality, throughout the interview, a strong sense of fearful resignation and a lack of openness to the future, perceived as something that could perhaps only get worse.

*Mmm... mmm, no, okay, it's not trying, because you get absorbed in what you have to do, so trying comes later, in that moment you do what you have to do. So... we started going to the check-ups, the counter-checks, and so on, so we were solely focused on doing... on understanding what it meant to not think about what we thought, that's the concept [...] Well, we were already expecting it, in some way we expected it, it was a confirmation. It's certainly not good news, but even in that case, I repeat, no despair, no feeling sorry for ourselves, no stuff. That's just how we are. Less about us, more about what needs to be done. So let's try to do the things that need to be done right away, let's not get lost in... sentimentality, let's put it that way. OK.*

*(father, ID 13)*

This narrative allows us to observe the total lack of emotional processing. The parent systematically avoids any emotional reference, taking refuge in mechanical and repetitive non-action, which serves a defensive function. The father's constant insistence on adoption thus reveals an emotional dulling, which does not express sadness, but rather suggests a weakened state. Through detachment and renunciation, he explores his own experiences.

4. Unresolved – Confused

The confused subcategory is characterized by a fragmented, incoherent, and sometimes wavering narrative, in which the parent struggles to maintain a logical or stable thread, easily loses focus, and demonstrates difficulty understanding, remembering, or defining the diagnosis and characteristics of their child.

In this group of participants, only one mother fell into this subcategory, and her case represents one of the clearest examples of unresolved situations. Her narrative is marked by severe cognitive and emotional confusion: the mother reports remembering almost nothing about the diagnostic process, struggles to understand what autism is, expresses unrealistic present and future perceptions of her daughter's condition, and alternates between moments of denial and alarm.

The following short but extremely dense excerpt clearly demonstrates the mother's difficulty constructing a coherent narrative about her daughter's diagnosis and her inability to assign meaning to it.

*I understand, that's it. I don't remember it anymore... uh... At the beginning... well... I don't remember the sensations anymore [...] sometimes I say, what is autism? I wonder, will she be crazy when she grows up? Is she crazy today? Who knows. This is what I ask myself: I don't understand who's crazy, who's autistic. Is someone who's crazy part of autism? Who knows. I don't know, I don't understand, and I don't know if I want to understand. [...] I don't know, I don't know. Have you ever seen those mental hospitals before? Crazy people, like this, crazy, throwing themselves down? I'm afraid of this. I'm afraid of this, and I say to myself, does this belong to this category? I don't know. I don't understand it […] I don't understand, sorry, I don't understand, I'm not a doctor and I don't know. Maybe I just can't understand it.*

*(mother, ID 16)*

This narrative represents the confused modality. This mother, in fact, appears trapped in a state of profound confusion, unable to process, remember, or place the experience in any meaningful framework. Confusion, in fact, is not only narrative, but represents a mode of emotional functioning in which the diagnosis remains an incomprehensible, unintegrated, feared, and continually avoided event.

5. Unresolved – Distorted

The distorted subcategory refers to those narratives in which the parent displays an unbalanced perception of their child's condition, characterized by unrealistic expectations, selective minimization of symptoms, or idealizing or devaluing interpretations that do not correspond to the clinical picture. Distortion affects both the understanding of the diagnosis and the representation of their child's current functioning and future possibilities. Therefore, there is often a difficulty recognizing the complexity of the clinical picture, thus favoring a narrative that protects the parent from confronting the most painful aspects of the diagnosis.

Within the group of participants in this study, only one mother was found to belong to this subcategory. Her interview fully represents the description just presented, in fact, it is characterized by a strong oscillation between minimizing symptoms, the realization of some of her daughter's skills, and a narrative that distinguishes between the actual diagnosis and a diagnosis on paper. The perception of the diagnostic picture thus appears to be filtered by a need for protection that leads to reinterpreting or reformulating clinical events, thus reducing their significance.

*No because she doesn't. She never isolated herself, she was never afraid of noises, she never… did any particular… strange things, let's say. The problem was that she didn't speak, that was the thing [...] Even if the real diagnosis… it's not autism level 3, that was done later to get support hours, otherwise we would have received almost nothing, but the real diagnosis is autistic traits, that is in fact the real diagnosis. When we then decided to get more hours, the doctor told us that we would find this written on paper, so from then on there is this transition, but it's not the real one. The problems are not that they don't exist, let's be clear, but it's not, for example… anyway, she's a sunny, cheerful girl, she likes being in company, she has many passions, anyway… the problems are there, she can't wait, she can't interact with her peers yet, that the only interaction she has, even if the teachers work with her, is through music, which is her passion, right? So she comes to you and suggests a song, in this sense. So… this [...] the problem is that everyone is different, what can I say about M. eh… it's a bit relative, because in my opinion… I'm not saying she's a unique case, a unique case not in the sense that she's special, but every child is different because, I repeat, there are some things I don't see in her about autism.*

*(mother, ID 15)*

6. Unresolved – Emotionally Overinvolved

Within this subcategory, unresolvedness manifests itself through overwhelming emotional involvement, which prevents the parent from organizing the experience into a coherent and reflective narrative. The pain, which is still too intense, is not symbolized but acted out within the interview, thus transforming the conversation into a predominantly expressive and containing space. Elements such as constant crying, difficulty constructing complete sentences, and the implicit need for emotional support signal a state of still acute suffering in which the diagnosis remains an unmentalized event. The interviewer is thus triangulated within the patient's pain and used as a container.

In this study, only one mother fell into this subcategory; her interview was marked by episodes of recurrent crying, prolonged pauses, and difficulty maintaining a linear narrative. In this case, the emotional overinvolvement emerged not only from the verbal content but also from the very form of the interview, which assumed a restraining function.

*Mother: “I felt… he didn't look at me, he didn't speak to me, he didn't say mommy or daddy. That's what he did to me…”*

*Interviewer: “What made you think something was wrong?”*

*Mother: “Yes… even though they said to wait until the child was three.”*

*Interviewer: “And you were worried?”*

*Mother: (crying) “Yes.”*

*Interviewer: “Do you remember how you felt at that time?”*

*Lady: “When A. was two years old, I never slept at night. Never. My head was always racing: I was thinking, ‘Is there something wrong? What should I do?’”*

*(Mother continues to cry)*

*(Mother, ID 20)*

The recurrent crying, insomnia, mental hyperactivity, and need for validation signal a still very acute suffering that hasn't found a stable symbolic form, in which the interviewer is involved as an emotional support figure.
