# Supplementary material for: The long-term psychological processing of an autism spectrum disorder diagnosis in parents
Source: Front Psychiatry. 2026 Apr 29;17:1782789. doi: 10.3389/fpsyt.2026.1782789 (PMC13168189; doi:10.3389/fpsyt.2026.1782789)
Supplement: Supplementary file 2 [file Table2.docx]

**Resolved and Unresolved subcategories.**

Specifically, for the Resolved pattern three subcategories are identified:

- Emotionally Oriented:

- Clear and recognizable presence of feelings in the interview;

- The ability and possibility to access and discuss one's feelings is a key element in dealing with the diagnosis;

- The parent does not appear to attempt to gain the interviewer's sympathy;

- The narrative appears clear and coherent.

- Action-oriented:

- The parent appears focused on the practical aspects of caring for their child;

- Predominance of statements regarding what needs to be done;

- Ability to organize caregiving routines appropriate to the condition and needs of their child's illness.

- Thought-oriented:

- Strong emphasis on cognitive processes by the parent;

- Active search for necessary information about the child and his or her disability;

- Tendency to integrate emotions and reflections, with responses that connect feelings to beliefs and meanings.

Within the Unresolved pattern, six subcategories were identified:

- Emotionally Overinvolved:

- The narrative appears full of pain and sadness;

- Attempts to gain/stimulate the interviewer's sympathy;

- Experiences of ongoing crisis in daily life are brought to light.

- Worried/Angry:

- Presence of strong anger in the responses to questions;

- Attempt to collude with the interviewer with one's own anger.

- Neutralizing:

- Total lack of memory or recognition of the presence of negative emotions related to one's child's diagnosis;

- A detailed account of events but devoid of an emotional component.

- Depressed/Passively Resigned:

- Strongly sad or passive tone during the interview;

- Subject appears entangled in the sadness of the experience;

- No signs of hope for the future appear in the account of events;

- Overinvolvement in the child's care.

- Distorted:

- Clearly distorted expectations regarding the child's current or future condition

- Perception Unbalanced, even with an idealization of benefits or costs.

- Confused:

- the interview structure appears inconsistent and contradictory,

- the parent often finds himself rambling or losing his train of thought,

- his positions are unclear and often oscillate between two opposite poles.

These subcategories were intended to reflect the predominance of a particular resolution or lack of resolution strategy in the experience of diagnosis, compared to the representation of one's child and the parental self (Wachtel and Carter, 2008; Scher-Censor et al., 2020)
